# Magnetic Resonance Imaging Findings in Childhood Epilepsy at a Tertiary Hospital in Kenya

**DOI:** 10.3389/fneur.2021.623960

**Published:** 2021-02-12

**Authors:** Pauline Samia, Nicholas Odero, Maureen Njoroge, Shem Ochieng, Jacqueline Mavuti, Sheila Waa, Samson Gwer

**Affiliations:** ^1^Department of Paediatrics and Child Health, Aga Khan University, Nairobi, Kenya; ^2^Department of Paediatrics, Nyamira County Hospital, Nyamira, Kenya; ^3^Department of Paediatrics, Kiambu Sub-County Hospital, Kiambu, Kenya; ^4^School of Medicine, Kenyatta University, Nairobi, Kenya; ^5^Department of Imaging and Diagnostic Radiology, Aga Khan University Hospital, Nairobi, Kenya; ^6^Gertrude's Children's Hospital, Nairobi, Kenya; ^7^Research and Evidence Unit, Afya Research Africa, Nairobi, Kenya

**Keywords:** epilepsy, child, Africa, magnetic resonance imaging, seizure

## Abstract

**Background:** Neuroimaging is important for determining etiology and guiding care in early childhood epilepsy. However, access to appropriate imaging in sub-Saharan Africa is modest, and as a consequence, etiological descriptions of childhood epilepsy in the region have been limited. We sought to describe MRI findings in children with epilepsy presenting to a tertiary hospital in Nairobi, Kenya, over a 6-year period of routine care.

**Materials and Methods:** We undertook a retrospective review of MRI findings of children aged between 0 and 18 years with a diagnosis of epilepsy presenting to the pediatric neurology department of Aga Khan University Hospital in Nairobi, Kenya, between January 2014 and July 2020. Over this period, the hospital had 1.5T MRI machines (GE1.5T Signa Excite and GE 1.5T Signa Explorer) and a 3T MRI machine (Philips 3T Ingenia). MRI images were independently reviewed by two study radiologists, and the findings were summarized and categorized into a study database. Related clinical and electroencephalographic (EEG) details were extracted from patient records. Categorical data analysis methods were applied to investigate for relationships between clinically relevant neuroimaging findings and key clinical and EEG observations.

**Results:** Over the study period, 288 children with a confirmed diagnosis of epilepsy had an MRI. They were of median age of 6 [interquartile range (IQR) 2–11] years. Ninety-five (33%) children had abnormal findings on imaging. The most common findings were encephalomalacia related to chronic infarcts (*n* = 18: 6.3%), cerebral atrophy (*n* = 11: 3.8%), disorders of neuronal migration (*n* = 11: 3.8%), periventricular leukomalacia (*n* = 9: 3.1%), and hippocampal sclerosis (*n* = 8: 2.8%). Findings related to infectious etiology were only observed in four children. Clinical comorbidity and inter-ictal epileptiform activity on EEG were independently associated with abnormal findings on imaging.

**Conclusion:** Up to a third of the children who underwent an MRI had a positive yield for abnormal findings. Imaging findings related to infectious etiologies were little observed in our cohort, in contradistinction to etiology studies in similar settings. At the time of the study, comorbidity and inter-ictal epileptiform activity on EEG were associated with abnormal findings on imaging and should be considered in informing prioritization for imaging in childhood epilepsy in this setting.

## Background

Recent studies estimate the prevalence of active convulsive epilepsy in Sub-Saharan Africa (SSA) at 7.0–14.8 per 1,000 people (1, 2). This is about double the estimate of 4.5–5.0 per 1,000 people in Europe (3). This pronounced burden is likely attributable to greater occurrence of central nervous system infections, perinatal insults, and traumatic brain injury (1, 4). These etiologies are more relevant in childhood, and with the recognized greater manifestation of genetic causes and structural abnormalities in early life, they altogether present an even considerable burden of epilepsy disease for children in SSA.

The burden of childhood epilepsy in SSA exists in the context of limited capacity for care, diagnostics, and treatment. Most countries in SSA have just a handful of pediatric neurologists and <1 physician per 1,000 (5, 6). Magnetic resonance imaging (MRI), the preferred imaging modality for epilepsy, is poorly accessible at 0.48 per million of the population even in the best of settings (7). Access to genetic testing services is very limited (4, 8). This lean diagnostic capacity is associated with an epilepsy treatment gap of up to 85% (2). Related to this is a poor description of etiology of childhood epilepsy in SSA. Early-onset epilepsy occurs in the critical time period of physical, psychosocial, and mental growth and development. Determining etiology and defining specific epilepsy syndromes where possible are important in guiding treatment and prognosis and optimizing the various aspects of developmental support in early life.

Imaging is crucial for making such determination. Neuroimaging is important in establishing etiology, providing prognosis, and planning appropriate care. The International League Against Epilepsy (ILAE) recommends neuroimaging for children younger than 2 years except in febrile seizures, where there is evidence for localization-related epilepsy with the exception of typically benign idiopathic epilepsy, in abnormal neurological examination, and in childhood epilepsy refractory to the initial two antiepileptic drugs (9). MRI is the preferred imaging modality of choice because of better anatomic definition and characterization of pathology (9). Computed tomography (CT) scan may not identify a number of abnormalities apparent on MRI but is more widely available, less costly, and mostly does not require sedation of the child. CT scan is easier to deploy in emergency situations and can detect tumors, malformations, stroke, and calcified and bone lesions (10). However, it has a relatively poor yield in identifying focal brain lesions (10).

Access to neuroimaging (MRI and CT scans) and neurophysiological testing [electroencephalography (EEG)] is typically concentrated in large urban centers in SSA (11, 12). Accordingly, there are few studies that have provided a description of imaging findings in children with epilepsy in SSA. We have not come across studies that detail their MRI findings in the context of a consistent MRI access protocol. There is need to have better descriptions of imaging findings in childhood epilepsy in SSA and to align imaging guidelines with considerations for the unique etiological profiles and the resource limitations in this setting. These observations can be complemented by EEG studies that are more accessible and could have a role in discerning and prioritizing candidates for imaging in the context of limited resources (13).

We sought to describe MRI findings of children with epilepsy seen at our center between 2014 and 2020 and to determine the association between clinical and EEG findings and clinically relevant findings on neuroimaging.

## Materials and Methods

We undertook a retrospective observational study of children with a primary diagnosis of epilepsy who underwent care and neuroimaging care at our center between January 2014 and July 2020.

### Study Setting

We conducted the study at the pediatric neurology department of the Aga Khan University Hospital (AKUH), in Nairobi, Kenya. AKUH is a tertiary hospital with a cosmopolitan catchment population that includes referrals from other parts of the country. There is no known endemic neurological disease in the immediate catchment population of Nairobi. The pediatric neurology department runs four clinics in a week, each with an approximate attendance of 15–20 patients. Approximately 70% of children seen at these clinics have epilepsy. Over the study period, this service was mostly supported by one neurologist and several pediatricians and pediatric registrars. The hospital has three MRI machines (GE1.5T Signa Excite, in place from 2012–2016; GE 1.5T Signa Explorer from 2016 to date; and Philips 3T Ingenia, in place since 2013 to date). The radiology department is supported by eight consultant radiologists, of whom two are neuroradiologists. The hospital's neurophysiology department has two 21-lead EEG machines and is supported by a neurophysiology team of three.

### Study Population

This study recruited all children aged 0–18 years who had been diagnosed with epilepsy according to the ILAE 2014 epilepsy guidelines and had undergone imaging at the hospital's imaging department. Children with seizures that were documented only in association with febrile illnesses were excluded from the study. The study did not consider imaging results undertaken at other centers outside the hospital.

### Standard Care

Children reviewed at the pediatric neurology presented as referrals from other pediatric clinics, hospitals, or the general outpatient department. The majority of clinic attendees were newly diagnosed cases. A diagnosis of epilepsy was made on history and examination as guided by ILAE guidelines, with the main working principle being history of unprovoked seizures occurring at least 24 h apart; extended after 2014 to consider diagnosis of an epilepsy syndrome and occurrence of only one seizure episode but with the possibility of suffering another seizure episode (14). Upon confirmation of a diagnosis of epilepsy, the children were started on antiepileptic drugs as guided by standard norms of treatment and in consideration of the child's age, existing comorbidity, cost of medication, and history of adverse effects. Assessment for comorbidities including attention deficit/hyperactivity disorder (ADHD), autism spectrum disorder (ASD), and developmental delay was done using clinical assessment criteria set out in Diagnostic and Statistical Manual of Mental Disorders, Fifth Edition (DSM 5), for ADHD and ASD and the Molteno Adapted Scale (validated for use in African populations) for developmental delay (14). Most children underwent routine EEG. MRI was considered for eligible children as per ILAE guidelines. Imaging was carried out using 1.5T MRI GE Signa Excite (2012–2016), 3T MRI Philips Ingenia (preferentially), and 1.5T MRI GE Signa Explorer (2017–2020). Choice of use of either a 1.5T or 3T machine was based on availability. The following sequences were applied: coronal T2 and FLAIR at 2-mm thickness and 1.5-mm spacing; axial T1, T2, FLAIR, T2 sagittal, all at 4-mm thickness and 2-mm spacing; diffusion-weighted imaging (DWI) at 4-mm thickness and 1-mm spacing; and coronal T1-weighted volume sequence at 1.2-mm thickness, no spacing. If contrast was considered necessary following review by radiologist and the attending neurologist, contrast was administered (gadodiamide, Omniscan, GE Healthcare, at 0.2 ml/kg), and post contrast T1 was acquired in all planes. All MRI images were reviewed and reported by a consultant radiologist, and the same was captured and stored in the hospital Picture Archiving and Communication System (PACS), available for review for up to 10 years from date of storage. Patients could choose to undertake imaging at another facility, in which case the results were documented in the clinical notes but the image films were retained by the patient.

### Study-Specific Procedures

Patients with a primary diagnosis of epilepsy who underwent MRI were identified from the pediatric neurology clinic attendance database and the hospital's PACS. Two study radiologists (SW and JM) independently reviewed the MRIs and entered their findings into a study database. These findings were reviewed for congruence, and if there were significant discrepancies, the images were re-presented to the radiologists for joint review and determination of consensus findings. On account of the format of data storage, the clinical data available from the clinic attendance database were limited to diagnosis and comorbidity details, age, gender, and a brief clinical summary. It was assumed that data on final diagnosis and comorbidity had taken into consideration clinical details on seizure semiology, investigations, physical examination findings, and other relevant clinical findings. EEG reports for patients who had monitoring at the hospital were retrieved and their details were abstracted into the study database, linking to the available imaging and clinical data.

### Data Management and Analysis

Patient data were deidentified during entry into the study database (Open Data Kit). Data analysis was undertaken using Intercooled STATA (version 13.0, StataCorp LP). The outcome of interest was findings on MRI. EEG report findings and clinical details were appropriately categorized for analysis. Kappa inter-rater statistics was applied to compare imaging findings between the two radiologists. Most of the data were categorical, and these were analyzed using chi-square test to determine significant associations with abnormal imaging findings. Multivariable logistic regression was applied to identify clinical and EEG findings that were independently associated with positive findings on MRI.

### Ethical Considerations

Approval to do the study was sought from the AKUH human ethics research committee and the National Commission for Science, Technology and Innovation (NACOSTI). Being a retrospective study, the data sought had been collected as part of routine care, no biological sample was collected, and the study was not investigating experimental or new protocols. As such, consent from individual study subjects was waived. Individual patient data were anonymized, and the study database was password protected and accessible only to the study investigators. There were defined minimal risks of confidentiality breach for patients involved in the study, but this was mitigated by appropriate data security measures and deidentification of personal data. The study was considered to have potential in improving the care for children with epilepsy.

## Results

**Figure 1:**
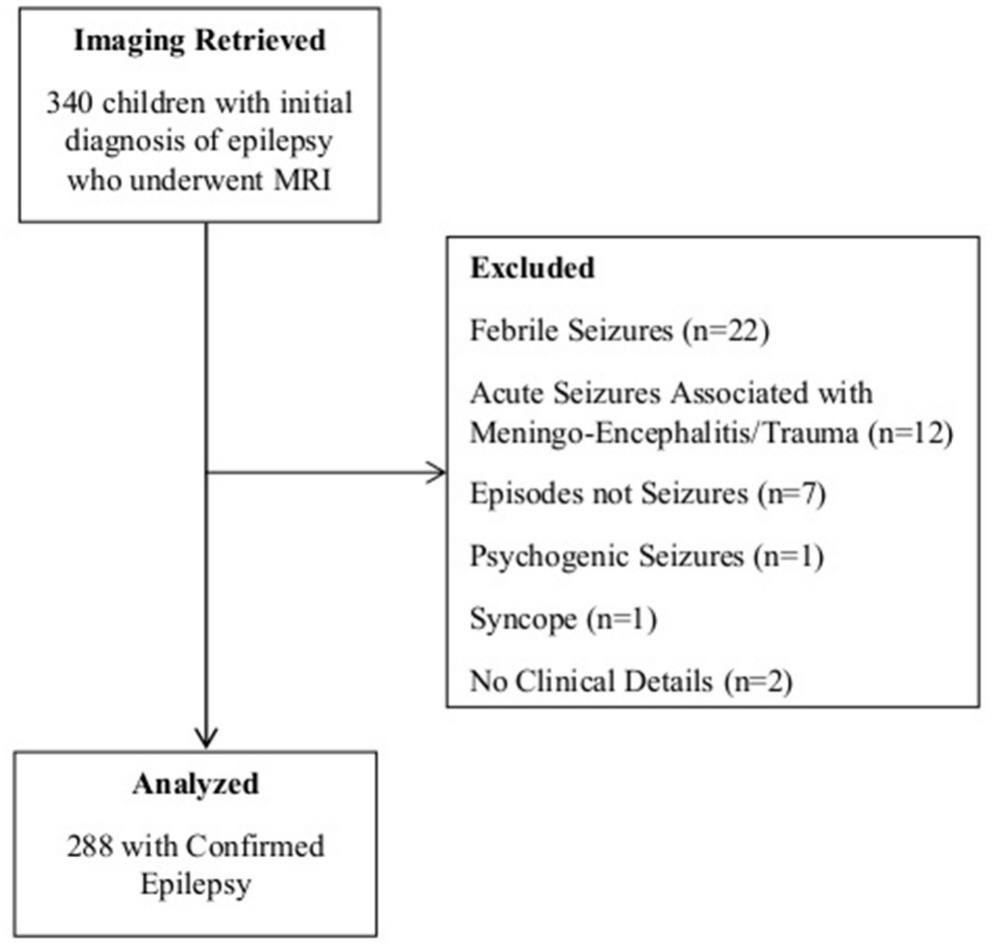
Study flowchart.

Between January 2014 and July 2020, 340 children seen at the pediatric neurology and developmental pediatrics departments with a working diagnosis of epilepsy underwent MRI at the hospital's imaging department. They were of median age of 6 [interquartile range (IQR) 2–11] years. Upon review of related clinical records, 52 children with alternative diagnoses were dropped from further analysis (Figure 1). Significant comorbidities were documented in 23% of the children (*n* = 66), the most common being global developmental delay or regression in 9% (*n* = 26); chronic headaches in 5% (*n* = 13); and ADHD, autism, and behavioral abnormalities in 3% (*n* = 8). Other comorbid conditions included speech delay, impaired vision, hemiplegia, hydrocephalus, microcephaly, diabetes, precocious puberty, and other conditions that were observed with smaller frequencies.

### Imaging Findings

The two radiologists (JM and SW) who reviewed the images had strong inter-rater agreement (kappa = 0.81). About a third of the children (*n* = 95) who underwent imaging had abnormal findings on imaging. The most common findings were encephalomalacia related to chronic infarcts, cerebral atrophy, disorders of neuronal migration, periventricular leukomalacia, and hippocampal sclerosis (Table 1). The identified disorders of neuronal migration included focal cortical dysplasia, pachygyria, lissencephaly, and subcortical heterotopia. Imaging findings of cerebral atrophy and periventricular leukomalacia were likely to be observed in younger children (<36 months). There were no consistent relationships between the various imaging findings and age. Only four children had findings suggestive of an infectious etiology: two with features of meningoencephalitis, one with tuberculoma, and one with a septic embolus with abscesses. Three patients had hippocampal post-ictal changes. Findings related to hemiconvulsion-hemiplegia, tuberous sclerosis, neurofibromatosis, and diffuse glioma were observed in two children each. Other significant abnormal findings were observed in singular frequency.

**Table 1:** Summary of imaging findings.

**Imaging findings**	**Frequency**	**Median age in months (IQR)**
Normal findings	193 (67.0%)	84 (36–144)
Encephalomalacia/chronic infarcts	18 (6.3%)	48 (12–156)
Cerebral atrophy	11 (3.8%)	12 (12–36)
Disorders of neuronal migration	11 (3.8%)	84 (5–120)
Periventricular leukomalacia	9 (3.1%)	24 (12–36)
Hippocampal sclerosis	8 (2.8%)	66 (30–156)
Choroidal and arachnoid cysts	7 (2.4%)	60 (48–72)
Hydrocephalus	4 (1.4%)	–
Others	27 (9.4%)	–

*IQR, interquartile range*.

### Electroencephalographic Findings

Forty-nine percent (*n* = 140) of the imaged children underwent routine EEG at the hospital's neurophysiology department. Thirty-two percent (*n* = 46) were documented to have generalized (22%) and focal (78%) interictal epileptiform discharges. Focal or diffuse slowing was reported in 17 (6%) children; 53% of them independent of inter-ictal epileptiform activity. Only seven children (5%) were reported to have asymmetrical background.

### Relationship Between Clinical, Electroencephalographic, and Imaging Findings

The presence of comorbidity and age ≤ 5 years were significantly associated with abnormal imaging findings (Table 2). Inter-ictal epileptiform EEG activity was also associated with abnormal findings on imaging. The other EEG findings were not significantly associated with abnormal imaging findings. On applying multivariable logistic regression, comorbidity and inter-ictal epileptiform EEG report finding were independently associated with abnormal imaging finding (Table 3).

**Table 2:** The relationship between electroencephalographic (EEG) and clinical features and abnormal imaging findings.

**Clinical and EEG findings**	**Abnormal imaging findings *n*/*N* (%)**	**Normal imaging *n*/*N* (%)**	**Univariable** **OR (95% CI)**	***P*-Value**
Age ≤ 5 years	52/133 (39%)	81/133 (61%)		
Age > 5 years	43/155 (28%)	112/155 (72%)	1.67 (1.01, 2.76)	0.04
Presence of comorbidity	35/66 (53%)	31/66 (47%)		
No documented comorbidity	60/222 (27%)	162/222 (73%)	3.04 (1.70, 5.47)	<0.01
Inter-ictal epileptiform EEG activity	19/45 (42%)	26/45 (58%)		
No inter-ictal epileptiform EEG activity	18/95 (19%)	77/95 (81%)	3.13 (1.39, 7.03)	<0.01
EEG slowing	7/17 (41%)	10/17 (59%)		
No documented EEG slowing	30/123 (30%)	93/123 (70%)	2.17 (0.75, 6.28)	0.14
Asymmetrical EEG background	3/7 (43%)	4 (57%)		
Symmetrical EEG background	34/133 (26%)	99/133(74%)	0.46 (0.10, 2.17)	0.31

*OR, odds ratio*.

**Table 3:** Multivariable logistic regression analysis of electroencephalographic (EEG) and clinical findings' association with abnormal imaging findings.

**Variables**	**OR** **(multivariable)**	**95% CI**	***P*-Value**
Comorbidity	3.603278	1.44, 9.01	<0.01
Age ≤ 5 years	1.308164	0.56, 3.06	0.54
Interictal_EEG	3.082167	1.36, 6.99	<0.01

*OR, odds ratio*.

## Discussion

MRI is fundamentally important and has become increasingly relevant in the evaluation and management of childhood epilepsy: supporting elucidation of etiology, syndrome description, prognostication, and determination of opportunities for definitive interventions (9). Identification of focal brain lesions on MRI has supported the scale-up of surgical interventions, delivering favorable outcomes for epilepsy conditions that were hitherto associated with refractory seizures and poor outcomes (9, 10, 15). In infectious pathologies, it is useful in discerning etiological agents and supporting targeted and adequate microbial care. MRI is essentially an indispensable tool in an optimally functioning center that cares for people with epilepsy. However, MRI practices are variable worldwide, particularly in SSA, on account of disparate resources, technical capacity, and infrastructure (7, 9). Identifying candidates for priority access is useful in informing judicious use where resources are limited.

Our study reveals a 33% yield in abnormal MRI findings out of the children with epilepsy who underwent imaging in our study. The most common imaging findings were encephalomalacia related to chronic infarcts, cerebral atrophy, disorders of neuronal migration, periventricular leukomalacia, and hippocampal sclerosis. A retrospective observational study of childhood epilepsy presentation at a tertiary hospital in South Africa with access to CT and MRI revealed abnormalities in 34% of the children, most common being features of perinatal hypoxic insult, disorders of neuronal migration, and neurocutaneous disorders (11). In this study, CT and MRI scans were prioritized for children with a history of trauma, with no obvious underlying cause, as part of a metabolic assessment, and in those with refractory epilepsy. A smaller case-control study in Tanzania revealed abnormalities in 29% of the children who had CT scans, describing brain atrophy and features of perinatal insults as the most common positive findings (16). Both studies show similar proportions of positive imaging yield. They also reveal an etiological profile that identifies perinatal insults as one of the most common findings: recognizing a preventable cause of epilepsy that calls for deliberate ameliorating interventions in maternal and newborn health. A Bhutan study that included adults and excluded children under the age of 5 years reported abnormal MRI findings in 81% of the patients, the most common findings being mesial temporal sclerosis and neurocyticercosis (17). Accordingly, different settings will have different observations in yield and profile description on imaging, underscoring the importance of a well-conducted childhood imaging description for our setting. Admittedly, some of our imaging findings are nonspecific in respect to etiological relationships, and observations of arachnoid and choroidal cysts were likely incidental. Significant observations of focal lesions in our study indicate the opportunity for surgical interventions in care in our setting, calling for the need for deliberate development of capacity for epilepsy surgery. Findings related to infectious etiology were not as commonly seen in our study as has been reported in other studies, an observation that demonstrates the heterogeneity of the SSA setting. Imaging is not complete in determination of infectious etiology, and it is possible that the observed imaging findings related to infectious etiology represent an underestimate of infectious etiology in the children in our study. However, these findings could also be on account of the declining impact of infectious etiologies on epilepsy morbidity in our setting, attributable to the success of public health interventions that include wide-scale water and sanitation programs, HIV/AIDs prevention and care programs, and universal *Haemophilus infleunzae* and *Streptococcus pneumoniae* vaccination programmes (18). Other studies suggesting greater occurrence of infectious etiology may have been biased to settings in which some of the infectious etiologies are endemic.

Comorbidity was independently associated with greater odds of positive imaging findings. We observed significant comorbidity in 23% of our children, a much lower finding than a previous observation of 54% neurobehavioral comorbidities in children with lifetime epilepsy in rural Coastal Kenya (19). Admittedly, ours is a retrospective study, and an assumption is being made that all the children were uniformly assessed for comorbidity, notwithstanding that such determination was not the primary aim of our study. Occurrence of comorbidity in epilepsy is an evolving clinical scenario, and our observations at a particular time period and age are not representative of lifetime prevalence. Further, it would be expected that children seen at a tertiary facility would have complicated epilepsies with associated greater comorbidity. However, like in most SSA settings, because of a suboptimal health system and a broken referral scheme, our hospital functions as a primary, secondary, and tertiary facility in one, and the profile of her catchment is not necessarily constituted only of patients requiring tertiary health services. It would certainly be useful to undertake a prospective study using consistent clinical criteria to determine the prevalence of comorbidity and etiology of childhood epilepsy in urban and rural facilities that likely have different disease profiles.

Inter-ictal epileptiform EEG activity was also independently associated with abnormal imaging findings. In our setting, EEG services to complement epilepsy care are becoming increasingly widely available, albeit with challenges in upholding quality in conduct and reporting. Considering their relatively lower costs, EEG services are scalable. It is feasible to consider applying EEG study findings and clinical findings of comorbidity to determine priority in access to MRI services where resources are limited.

Ours was a retrospective study, and it has attendant weaknesses associated with this design. These include missing data from EEG and clinical observations. We omitted imaging and EEG data from studies not done at our center because of our inability to confirm the validity of the reported findings, potentially introducing selection bias in our observations. With a prospective design, we would have had access and opportunity to review MRI images from other centers, allowing for a more complete description. We also acknowledge the risk of misclassification bias in our assessment of imaging, clinical, and EEG findings. To mitigate this, we reviewed imaging and EEG records for accuracy.

Our study included a sizable number of children with epilepsy who underwent MRI in the context of a consistent imaging protocol. It provides a description of imaging findings of children with epilepsy from a cosmopolitan background in this setting, representing greater generalizability in findings than has been provided by other studies in the same setting. Overall, we provide a description of imaging findings in childhood epilepsy in SSA, providing greater insights on etiology of childhood epilepsy in our setting and defining the opportunity for surgical interventions and promoting public health interventions that facilitate access to optimal skilled delivery services.

## Data Availability Statement

The raw data supporting the conclusions of this article will be made available by the authors, without undue reservation.

## Ethics Statement

The studies involving human participants were reviewed and approved by Human research ethics committee of Aga Khan University. Written informed consent from the participants' legal guardian/next of kin was not required to participate in this study in accordance with the national legislation and the institutional requirements.

## Author Contributions

PS, SW, JM, and SG were involved in designing the study, collecting and analyzing the results, and drafting the final manuscript. SO, NO, and MN were involved in designing the data collection tools, collecting the clinical data, reviewing the EEG reports, analyzing the results, and drafting the final manuscript. All authors contributed to the article and approved the submitted version.

## Conflict of Interest

The authors declare that the research was conducted in the absence of any commercial or financial relationships that could be construed as a potential conflict of interest.
